# Supplementary material for: Reduction of Fat to Muscle Mass Ratio Is Associated with Improvement of Liver Stiffness in Diabetic Patients with Non-Alcoholic Fatty Liver Disease
Source: J Clin Med. 2019 Dec 9;8(12):2175. doi: 10.3390/jcm8122175 (PMC6947171; doi:10.3390/jcm8122175)
Supplement: Supplementary File 1 [file jcm-08-02175-s001.pdf]

Supplemental Table. The difference of Characteristic between study participants with or without performing follow-up.

	**No Follow-up**	**Follow-up**	***p* Value**
N	129	117	-
Men	81 (62.8)	59 (50.4)	0.054
Age, year	65.1 (12.4)	63.5 (12.2)	0.318
Duration of diabetes, years	16.6 (18.5)	12.0 (9.6)	0.029
Body mass index, kg/m^2	25.1 (4.3)	25.4 (4.4)	0.767
Appendicular skeletal muscle mass, kg	20.4 (4.1)	19.0 (4.8)	0.938
Skeletal muscle index, kg/m^2	7.3 (1.2)	7.1 (1.2)	0.243
Body fat mass, kg	19.8 (8.3)	21.2 (8.2)	0.157
Body fat percentage, %	29.3 (9.1)	31.1 (8.2)	0.131
Fat to muscle ratio	0.8 (0.3)	0.9 (0.3)	0.167
Systolic blood pressure, mmHg	132.7 (16.0)	134.0 (19.8)	0.500
Diastolic blood pressure, mmHg	77.9 (9.2)	79.1 (11.6)	0.782
Platelet count, × 10^9/L	218.4 (56.5)	218.6 (53.9)	0.970
Hemoglobin A1c, %	7.4 (1.1)	7.5 (1.1)	0.593
Hemoglobin A1c, mmol/L	57.7 (12.4)	58.4 (12.4)	0.593
Aspartate aminotransferase, IU/L	26.8 (13.0)	29.8 (18.6)	0.145
Alanine aminotransferase, IU/L	28.3 (19.9)	34.9 (30.0)	0.040
Gamma-glutamyl transferase, IU/L	44.0 (67.0)	47.1 (46.4)	0.675
Ferritin, ng/mL	144.5 (153.0)	126.1 (116.6)	0.340
Type 4 collagen 7 S, ng/mL	5.0 (1.2)	4.8 (1.0)	0.512
Hyaluronic acid, ng/mL	107.3 (218.8.8)	85.0 (134.2)	0.284
Fib-4 index	1.76 (01.06)	1.67 (0.84)	0.448
Controlled attenuation parameter, dB/m	260.2 (71.3)	273.4 (53.5)	0.113
Liver stiffness measurement, kPa	6.3 (4.2)	6.3 (3.4)	0.931

Continuous variables were presented as the mean (standard deviation; SD) and categorical variables were presented as number (percentage). The paired t test (continuous variable) and Wilcoxon signed-rank test (categorical variable) were performed to identify the statistical differences between baseline and one year after.
